# Electron beam therapy at extended source‐to‐surface distance: a Monte Carlo investigation

**DOI:** 10.1120/jacmp.v9i4.2811

**Published:** 2008-10-24

**Authors:** Tuathan P. O'Shea, Mark J. Foley, David Rajasekar, Patrick A. Downes, Wil van der Putten, Margaret Moore, Andrew Shearer

**Affiliations:** ^1^ Medical Physics Group, School of Physics National University of Ireland Galway Galway Ireland; ^2^ Department of Medical Physics and Bioengineering University Hospital Galway Galway Ireland; ^3^ Department of Information Technology National University of Ireland Galway Galway Ireland

**Keywords:** electron beam, extended SSD, output factor, Monte Carlo, BEAMnrc

## Abstract

Electron‐beam therapy is used to treat superficial tumors at a standard 100 cm source‐to‐surface distance (SSD). However, certain clinical situations require the use of an extended SSD. In the present study, Monte Carlo methods were used to investigate clinical electron beams, at standard and non‐standard SSDs, from a Siemens Oncor Avant Garde (Siemens Healthcare, Erlangen, Germany) linear accelerator (LINAC). The LINAC treatment head was modeled in BEAMnrc for electron fields 5 cm in diameter and 10×10 cm, 15×15 cm, and 20×20 cm; for 6 MeV, 9 MeV, and 12 MeV; and for 100 cm, 110 cm, and 120 cm SSD. The DOSXYZnrc code was used to calculate extended SSD factors and dose contributions from various parts of the treatment head.

The main effects of extended SSD on water phantom dose distributions were verified by Monte Carlo methods. Monte Carlo–calculated and measured extended SSD factors showed an average difference of ±1.8%. For the field 5 cm in diameter, the relative output at extended SSD declined more rapidly than it did for the larger fields. An investigation of output contributions showed this decline was mainly a result of a rapid loss of scatter dose reaching the $d_{\text{max}}$ point from the lower scrapers of the electron applicator. The field 5 cm in diameter showed a reduction in dose contributions; the larger fields generally showed an increased contribution from the scrapers with increase in SSD. Angular distributions of applicator‐scattered electrons have shown a large number of acute‐angle electron tracks contributing to the output for larger field sizes, explaining the shallow output reduction.

PACS numbers: 87.53.Wz, 87.53.Vb, 87.53.Hv

## I. INTRODUCTION

Electron‐beam therapy is used in the treatment of superficial tumors and to administer boost doses to tumor sites. It is usually performed at the standard source‐to‐surface distance (SSD) of 100 cm. At this distance, the characteristics of the radiation beam such as percentage depth dose, off‐axis profiles, and output factors are known. In certain situations, anatomic constraints may dictate the use of an extended SSD (for example, 110 cm SSD). In the treatment of the lateral neck region, for instance, a patient's shoulder may obstruct the positioning of the electron applicator. In the clinic, 9 MeV and 12 MeV extended SSD treatment is routinely used in head‐and‐neck cases. At extended SSD, the output and dose profile characteristics of the radiation beam are altered, leading to uncertainty in treatment. The effects of nonstandard SSD on machine output and dose distributions must be assessed to ensure accurate patient treatment.^(1)^

The main effects of extended SSD on clinical electron‐beam characteristics have been outlined by Cygler et al.^(2)^ and Khan.^(3)^ These changes to the radiation beam characteristics are mainly a result of the variations in scattered electron tracks at various levels. With the availability of Monte Carlo codes such as BEAM,^(4)^ and the ability to extract detailed particle history information, it is possible to investigate how the electron scattering off each component of the linear accelerator (LINAC) treatment head affects dose distributions and beam output.

Electron‐beam output does not follow the inverse square law (ISL) as photon beam output does. Two methods of output correction are typically used—namely, the effective SSD method^(3)^ and the virtual SSD method.^(5)^ The dose deposited at $d_{\max}$ depends on the number of electron tracks traversing the measuring volume. Some electrons come directly from the source, traversing the scattering foils and dose chambers, scattering only in the intervening air (the direct component of the beam). The remainder are scattered by the photon jaws, the multileaf collimator, and the scrapers of the electron applicator (the indirect component of the beam). Which of these components contributes most to the change in output depends on energy, collimator design, field size, and SSD.

The present work uses Monte Carlo simulation methods to report on the influence of the direct and indirect components of the electron beam from a Siemens Oncor (Siemens Healthcare, Erlangen, Germany) LINAC on various beam parameters.

## II. METHODS

### A. Measurements

Central axis percentage depth dose and off‐axis profiles were measured for fields 5 cm in diameter and 10×10 cm, 15×15 cm, and 20×20 cm at 100 cm SSD in a water phantom. These measurements were performed using a $1\;{\text{mm}}^{2}$ p‐type Si diode detector 2.5 μm thick (PTW, Freiburg, Germany) and a 0.3 cm^3^ thimble chamber (PTW) respectively.

Extended SSD factors were measured for 6 MeV, 9 MeV, and 12 MeV using a Roos chamber (PTW) with an active volume of 0.35 cm^3^. This device was calibrated (NPL, Teddington, U.K.) with a reported uncertainty of 1.6%. Measurements were taken at the depth of maximum dose, $d_{\max}$, for each setup—typically, 1.3 cm, 2.05 cm, and 2.7 cm for 6 MeV, 9 MeV, and 12 MeV respectively.

All measurements and subsequent conversions to dose were performed using Institute of Physics and Engineering in Medicine Code of Practice.^(6)^

### B. Monte Carlo calculations

#### B.1 LINAC modeling

**Figure 1:**
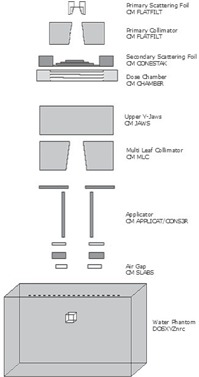
Schematic of the Siemens Oncor (Siemens Healthcare, Erlangen, Germany) treatment head, showing component modules used for BEAMnrc simulations.

BEAMnrc^(7)^ was used to model the Siemens Oncor LINAC treatment head using manufacturer‐provided specifications. Fig. 1 shows the treatment head components included in this study.

The applicator, which is positioned between z=56cm and z=95cm from focus, comprises 5 main parts. For the purposes of this study, they are designated scrapers 1 – 5 respectively, with scraper 5 being the nearest to the phantom surface. The applicator was modeled using the APPLICAT component module (CM) in BEAMnrc. The APPLICAT CM can handle only square or rectangular geometries, and therefore, for the applicator 5 cm in diameter, scrapers 1 – 4 were modeled with APPLICAT, and the circular aperture of scraper 5 was modeled with CONS3R CM.

The electron source, incident on the primary scattering foil, was modeled as a Gaussian electron beam using ISOURC=19. Each CM in the LINAC model was assigned a LATCH bit. This particle tracking variable allows detailed particle interaction history to be extracted upon completion of the simulation.

The main BEAMnrc input parameters used were ECUT and PCUT, valued at 0.521 and 0.01 MeV respectively. The electron range rejection (ECUTRR) variance reduction technique was used with an ESAVE_GLOBAL of 1.0 MeV to avoid simulating electrons that did not affect the phase‐space output significantly. Thus, any electron below 1.0 MeV was estimated to determine whether its range was short enough to terminate its transport.^(8)^

#### B.2 Dose calculations

The accelerator model generated with BEAMnrc was compiled as a shared library and run by the DOSXYZnrc^(9)^ user code. This approach eliminated the need to store intermediate phase‐space data, which requires large amounts of disk space. In the shared‐library method, the entire treatment head transport and water phantom dose calculation is performed in a single simulation step by DOSXYZnrc.^(10)^

DOSXYZnrc was used for dose calculations in water phantom models. The LINAC head model configured in BEAMnrc was validated by comparing measured and DOSXYZnrc‐calculated percentage depth dose and in‐plane dose profiles. Model fine‐tuning was necessary for agreement between measurement and calculations. This fine‐tuning involved adjustments to the electron source, energy, and full width at half maximum (FWHM) values. The source model producing the best agreement with measured data was chosen to be the fine‐tuned model.^(11)^

The number of histories in the DOSXYZnrc input were specified to produce a statistical uncertainty in calculated dose approaching 1%. This choice involved simulation of the order of $2\times 10^{7}$ to $2\times 10^{8}$ primary particle histories, depending on energy, field size, and SSD.

The water phantom model used for extended SSD factor calculations at $d_{\max}$ is shown in Fig. 1. A 0.5 cm^3^ voxel was centered at $d_{\max}$ to represent the detector used for measurements. Extended SSD factors were calculated using the calculated dose in that volume. Dose components were extracted using the LATCH filter.^(9)^

## III. RESULTS

### A. Percentage depth dose and dose profiles

**Figure 2:**
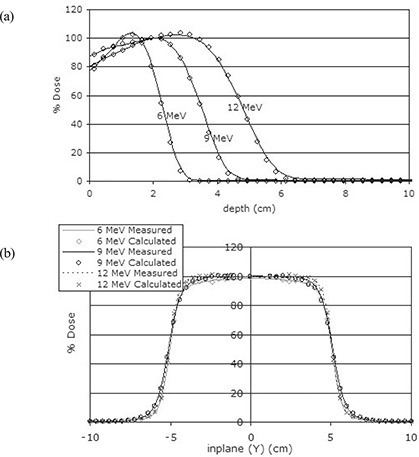
Comparison of measured and calculated (a) percentage depth dose curves (solid lines=measurement, circles=calculation), and (b) dose profiles for the 10×10 cm field size at 100 cm source‐to‐surface distance.

The model fine‐tuning process resulted in peak energies of 6.33 MeV, 9.20 MeV, and 12.25 MeV for 6 MeV, 9 MeV, and 12 MeV electron beams respectively. The FWHM was set to 0.103 cm. Once the electron models had been fine‐tuned, comprehensive benchmarking was performed by comparison of measured and calculated percentage depth dose and dose profiles in water. Calculated depth dose curves and dose profiles were plotted using STATDOSE.^(12)^ Data were normalized to $d_{\max}$. Fig. 2 shows a comparison of the percentage depth dose curves and in‐plane profiles at $d_{\max}$. Differences were within 2% over the entire range of configurations in this study.

**Figure 3:**
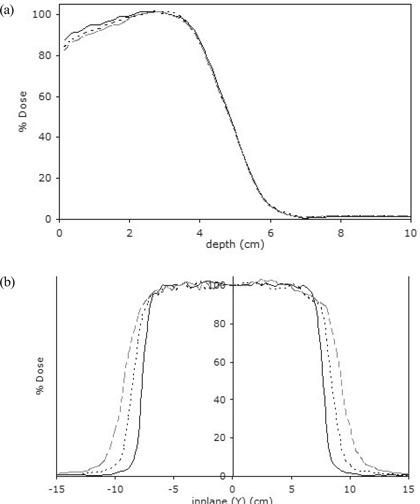
Effects of extended source‐to‐surface distance (SSD) on Monte Carlo–calculated (a) percentage depth dose curves and (b) dose profiles—in this case, for a 12 MeV electron beam and 15×15 cm field size (solid line=100 SSD; short‐dash line=110 SSD; long‐dash line=120 SSD).

**Figure 4:**
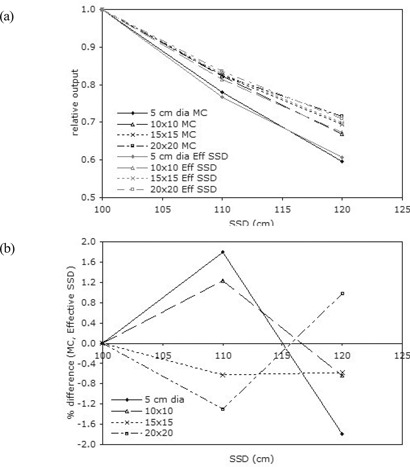
(a) Monte Carlo and effective source‐to‐surface distance (SSD) relative outputs for 9 MeV electron beam. (b) Percentage differences between Monte Carlo and effective SSD.

BEAMnrc‐simulated electron beams show results similar to measured and published results.^(2)^ ^,^ ^(13)^ ^,^ ^(14)^ Minimal effects of extended SSD on the characteristic parameters (dose buildup, $d_{\max}$, and dose falloff) of the depth dose curves were observed, represented by a dose reduction in the buildup region. The dose falloff region remained relatively constant with increase in SSD [Fig. 3(a)]. Relative output declined with increase in SSD. For the smallest field, defined by the applicator 5 cm in diameter, relative output declined more rapidly than it did for the larger fields. For the larger fields (10×10 cm, 15×15 cm, 20×20 cm), the relative output reductions with increase in SSD were comparable. Relative outputs show obvious deviations from the ISL with extended SSD for some configurations. For 12 MeV, the larger fields follow the ISL quite well. However, for the field 5 cm in diameter, significant deviations occur. For 6 MeV, all fields show large deviations from the ISL. Monte Carlo is in good agreement with the effective SSD method, which is often used to correct for nonstandard SSD electron beams in clinical calculations (Fig. 4), within 2% over the entire range of the study.

The effects of extended SSD on transverse beam profiles were verified as loss of flatness and increase in the penumbra [Fig. 3(b)].

### B. Calculations of $d_{\max}$

Table 1 compares measured and calculated extended SSD factors for the various electron beams and applicators included in the study. Generally, a difference of less than 2% was observed between measurement and calculation.

**Table 1:** Comparison of calculated and measured extended source‐to‐surface distance (SSD) factors

*Energy (MeV)*	*Field size (cm)*	*SSD (cm)*	*Measured* ^a^	*Calculated*	*Calculation Uncertainty (%)*	*Difference (%)*
6	5 (diameter)	100	1.000	1.000	1.1	˜
		110	0.734	0.721	1.3	1.8
		120	0.516	0.526	1.5	1.8
	10×10	100	1.000	1.000	0.6	˜
		110	0.800	0.786	0.6	1.7
		120	0.651	0.639	0.7	1.9
	15×15	100	1.000	1.000	0.8	˜
		110	0.827	0.840	0.8	1.5
	20×20	120 100	0.690 1.000	0.704 1.000	0.9 0.9	2.0 ˜
		110	0.837	0.822	1.6	1.8
		120	0.701	0.688	1.1	1.9
9	5 (diameter)	100	1.000	1.000	0.8	˜
		110	0.783	0.781	0.9	0.3
		120	0.602	0.596	1.0	1.0
	10×10	100	1.000	1.000	0.9	˜
		110	0.816	0.823	1.0	0.9
		120	0.674	0.670	1.1	0.6
	15×15	100	1.000	1.000	1.3	˜
		110	0.833	0.824	1.1	1.0
		120	0.698	0.695	1.2	0.4
	20×20	100	1.000	1.000	1.0	˜
		110	0.836	0.825	1.1	1.4
		120	0.707	0.715	1.2	1.2
12	5 (diameter)	100	1.000	1.000	0.9	˜
		110	0.796	0.797	1.0	0.2
		120	0.641	0.647	1.1	0.9
	10×10	100	1.000	1.000	0.9	˜
		110	0.826	0.828	0.9	0.3
		120	0.690	0.698	1.0	1.2
	15×15	100	1.000	1.000	0.9	˜
		110	0.838	0.833	1.0	0.6
		120	0.706	0.703	1.0	0.5
	20×20	100	1.000	1.000	1.0	˜
		110	0.840	0.818	1.1	2.7
		120	0.711	0.705	1.2	0.8

a ^a^ Measurement uncertainty was reported at 1.6%.

Monte Carlo–calculated extended SSD factors were decomposed into contributions from the various treatment head parts: the direct factor attributable to dose scored by direct particles (that is, electrons emanating directly from the source, traversing the scattering foils and dose chambers, scattering only in the intervening air) and the indirect factor attributable to dose from head‐scattered particles. The indirect factor was further decomposed into the separate contributions from the X–Y jaws and each applicator scraper. Output components were analyzed relative to each total extended SSD factor at a given SSD.

The direct component was found to be the major contributor in all cases. For 6 MeV and 9 MeV, this contribution increased with increase in field size, and the indirect component decreased. For 12 MeV, the same case held, except for the 10×10 cm field, which showed a smaller direct component as compared with the field 5 cm in diameter. The direct component appears to follow the ISL quite well, especially for 9 MeV and 12 MeV. Contributions from the applicator scrapers varied with field size. Scrapers 3 and 4 (that is, the middle trimmer) and 5 (the lowest trimmer) were, in most cases, the principal contributors to the applicator dose component. The X–Y jaws component show that, for higher energies (9 MeV and 12 MeV), the contribution of the jaws decreases with increase in field size.

At extended SSD, it was found that, for the smaller fields, particularly the field 5 cm in diameter, direct and indirect components generally showed trends opposite to those of the larger fields (especially up to 110 cm SSD). For the field 5 cm in diameter, the direct component tended to increase with extended SSD, and the indirect component decreased. For the larger fields, the direct component tended to decrease, and the indirect component, to increase. For all energies, the X–Y jaw output component remained relatively constant with increase in SSD.

**Figure 5:**
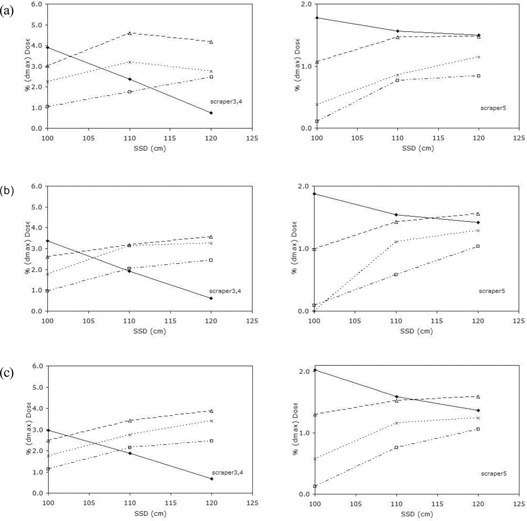
Variation of Monte Carlo output contributions for (a) 6 MeV, (b) 9 MeV, and (c) 12 MeV from applicator scrapers 3 – 5 with extended source‐to‐surface distance (SSD). Filled diamonds=5 cm diameter; triangles=10×10 cm field; crosses=15×15 cm field; squares=20×20 cm field.

For all energies and SSDs, the 15×15 cm applicator (as compared with the other applicators) showed the largest scraper 1 contribution to output. For scraper 2, the absolute contribution to output was lower with lower energy. With extended SSD, the output contributions from scrapers 3, 4, and 5 clearly showed an opposite trend between the field 5 cm in diameter and the larger fields (Fig. 5). For the field 5 cm in diameter, the contributions decreased with increase in SSD; for the larger fields, contributions increased with increase in SSD. It was also noted that the lower‐energy beam showed an increased output contribution from scrapers 3 and 4, and that scraper 5 contributions showed an increase with higher energy.

### C. Electron angular distributions

**Figure 6:**
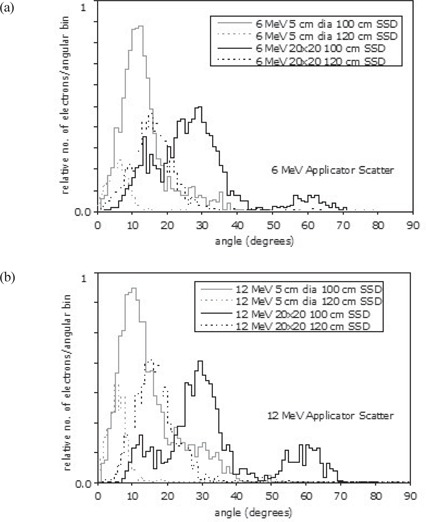
Angular distributions of applicator‐scattered electrons crossing a plane representing the transverse cross‐section of the $d_{\max}$ voxel for (a) 6 MeV and (b) 12 MeV, fields of 5 cm in diameter and 20×20 cm at 100 cm and 120 cm source‐to‐surface distance (SSD).

Fig. 6(a),(b) shows the angular distributions of electrons scattered from the applicators 5 cm in diameter and 20×20 cm for 6 MeV and 12 MeV at 100 cm and 120 cm SSD. (Similar results were found for 9 MeV.) This analysis was performed in BEAMDP^(15)^ using phase‐space data from the LINAC model. The scoring field was configured to represent the transverse cross‐section of the 0.5 cm^3^ $d_{\text{max}}$ voxel. Angular distributions were plotted as the number of electrons per angular bin against the scattering angle in degrees.

At 100 cm SSD, the large peak around 12 degrees for the applicator 5 cm in diameter represents electron tracks from the lower scrapers. At 120 cm SSD, many of these tracks no longer contribute to the output. The distribution shifts toward smaller angles, and the number of electron tracks declines significantly. The 20×20 cm applicator distributions clearly show tracks from the final scrapers—that is, the peak around 30 degrees (attributable to scrapers 3 and 4) and the peak around 60 degrees (attributable to scraper 5). At 120 cm SSD, the distributions lose the large‐angle tracks and shift toward more acute angles. The absolute number of electron tracks does not decline, however. A large number of acute‐angle electron tracks therefore influence the output at 120 cm SSD.

## IV. DISCUSSION

**Figure 7:**
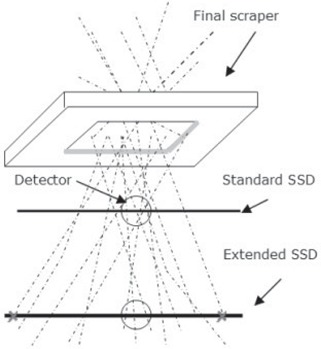
Diagram of electron tracks through the final scraper of the electron applicator. SSD=source‐to‐surface distance.

Monte Carlo–calculated depth dose and dose profiles at extended SSD agree with measured and published data.(2,13,14) The relative output at extended SSD for the smallest field was also found to decrease more rapidly than that for the larger fields.(14) For the field 5 cm in diameter, the X–Y jaws and applicator scrapers are closer to the beam central axis, and scattered radiation therefore affects the dose at $d_{\max}$ to a greater extent. These electrons were scattered at angles such that they cannot reach the detector at extended SSD (Fig. 7). Measurement of this type uses the so‐called broad beam geometry.(3) For the larger fields, the treatment head collimators are further from the beam central axis, resulting in fewer scattered electrons influencing dose at $d_{\max}$. However, as SSD increases, electrons scattered at relatively acute angles can reach the $d_{\max}$ point.(14) For the applicator 5 cm in diameter, relative output is affected by increased distance (ISL) and loss of scattered radiation. For larger fields, on the other hand, output is affected by ISL, but more scattered electrons reach the detector at $d_{\max}$.

The results of decomposition of Monte Carlo extended SSD factors into direct and indirect components showed results similar to those in previous studies.^(16)^ ^,^^(17)^ The direct component has been found to be approximately 90% of the dose at $d_{\max}$ for large fields from a Siemens MD2 LINAC.^(17)^ In the present work, the direct component ranged from approximately 90% for the smallest field to approximately 95% for the largest field. As with the study by Zhang et al.,^(17)^ for a given field, the percentage dose component from the Oncor's jaws remains relatively constant with SSD. In the present study, the applicator contributed more to $d_{\max}$ dose than the jaws did under most configurations. For the MD2 LINAC study, it was found that about 50% of the scattered dose component came from the jaws; the other 50% came from the applicator scrapers.^(17)^ Scrapers 3 and 4 (the middle trimmer) were found to contribute the most to the applicator scatter component. That finding is consistent with a previous study of a Siemens electron applicator.^(18)^

Analysis of the variation of Monte Carlo 6 MeV, 9 MeV, and 12 MeV output contributions by applicator scrapers 3, 4, and 5 with extended SSD showed different trends between the field 5 cm in diameter and the larger fields (Fig. 5). This observation explains the more rapid output reduction for the field 5 cm in diameter as compared with the larger fields. It is caused by a reduction in scattered dose contributions from scrapers 3, 4, and 5 with increase in SSD. The angular distributions of applicator‐scattered electrons (Fig. 6) also verify these findings. The relative number of electron tracks from the applicator contributing to the output at extended SSD is seen to be reduced significantly for the field 5 cm in diameter. However, for the larger fields, this reduction in electron tracks is not seen, because the acute‐angle electrons that cannot reach the detector at standard SSD contribute to the output at extended SSD.

## V. CONCLUSIONS

BEAMnrc was used to create an accurate model of a Siemens Oncor treatment head for electron fields at standard and non‐standard SSD. There was good agreement (to within 2%) between measured and calculated extended SSD factors. Decomposing Monte Carlo–calculated factors and analysis of scattered electron angular distributions helped in understanding the variation in output with field size and SSD. The dose contributed by electron scattering off the lower applicator scrapers decreased with increase in SSD for the applicator 5 cm in diameter, and the angular distribution showed only a small number of acute‐angle electron tracks reaching the detector at extended SSD. However, for the larger fields, the dose component from the lower scrapers increased with increase in SSD, and angular distributions showed that a larger number of acute‐angle electron reached the detector at extended SSD.

The present Monte Carlo study has helped in understanding some of the uncertainty—namely, variation in output and dose distributions—surrounding extended electron treatment for one of our Siemens Oncor LINACs. Because electron‐beam characteristics are a complex function of LINAC head design, these results may not apply to other LINAC models, and similar studies of other machines may be worthwhile.
